# Supplementary material for: Role of Ge:As ratio in controlling the light-induced response of a-GexAs35−xSe65 thin films
Source: Sci Rep. 2014 Feb 7;4:4029. doi: 10.1038/srep04029 (PMC3916963; doi:10.1038/srep04029)
Supplement: Supplementary Information — Supplementary Info File #1 [file srep04029-s1.pdf]

Supporting Information

Role of Ge:As ratio in controlling the light-induced response of a-$Ge_xAs_{35-x}Se_{65}$ thin films

Pritam Khan[1], H. Jain[2] and K.V. Adarsh[1*]

[1]*Department of Physics, Indian Institute of Science Education and Research, Bhopal 462023, India.*

[2]*Department of Materials Science and Engineering, Lehigh University, Bethlehem, Pennsylvania 18015, USA*

Supplementary figures

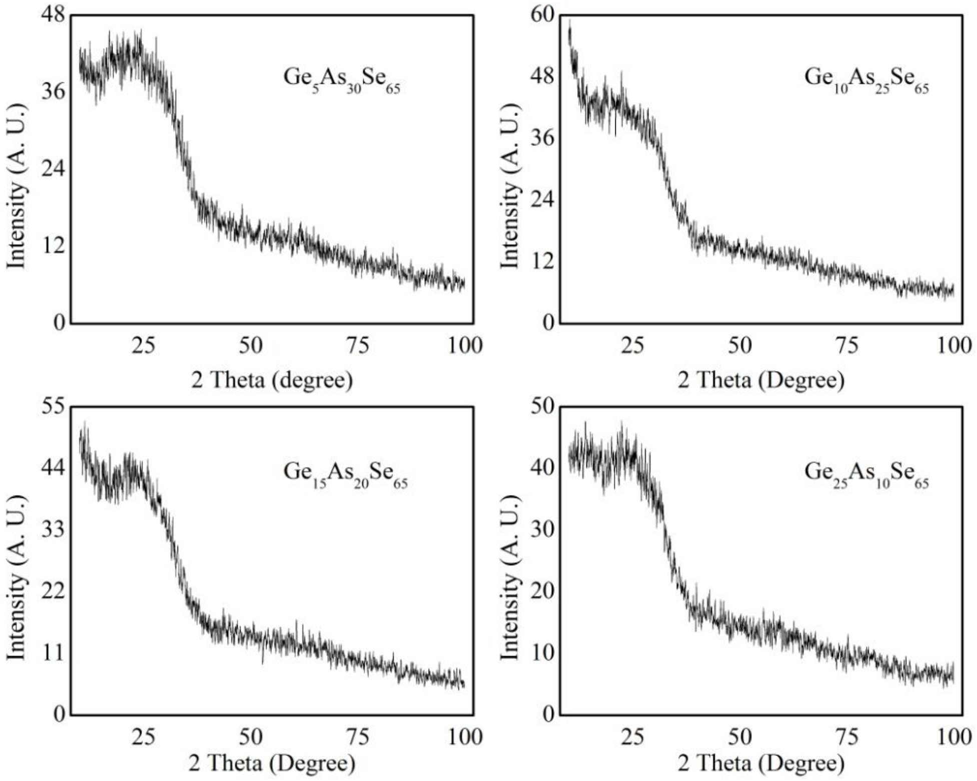

Figure S1. XRD images of $Ge_xAs_{35-x}Se_{65}$ thin films. It is quite evident from the figure that films are amorphous in nature.

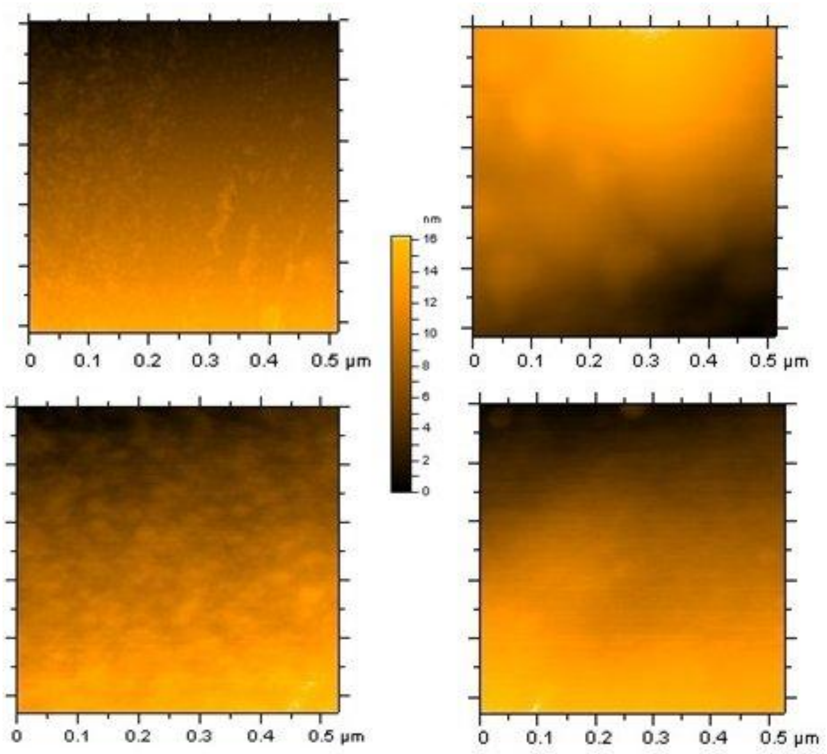

Figure S2. AFM images of $Ge_xAs_{35-x}Se_{65}$ thin films. Figures clearly indicate that film surface is homogeneous with no cracks or defects.

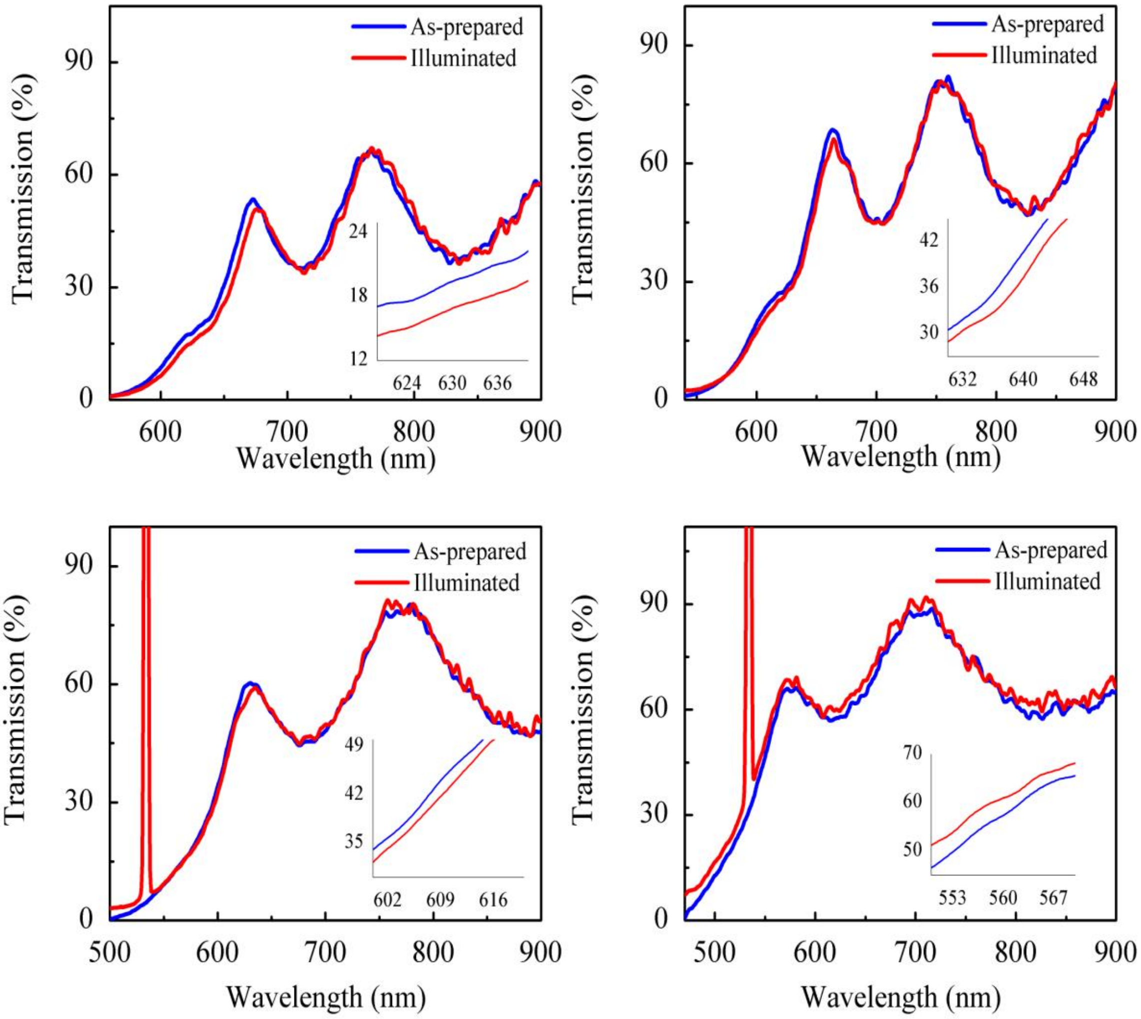

Figure S3. Transmission spectra of (a) a-$Ge_5As_{30}Se_{65}$ (b) a-$Ge_{10}As_{25}Se_{65}$ (c) a-$Ge_{15}As_{20}Se_{65}$ (d) a-$Ge_{25}As_{10}Se_{65}$ thin films in as-prepared and illuminated state. Inset shows expanded region near to bandgap.

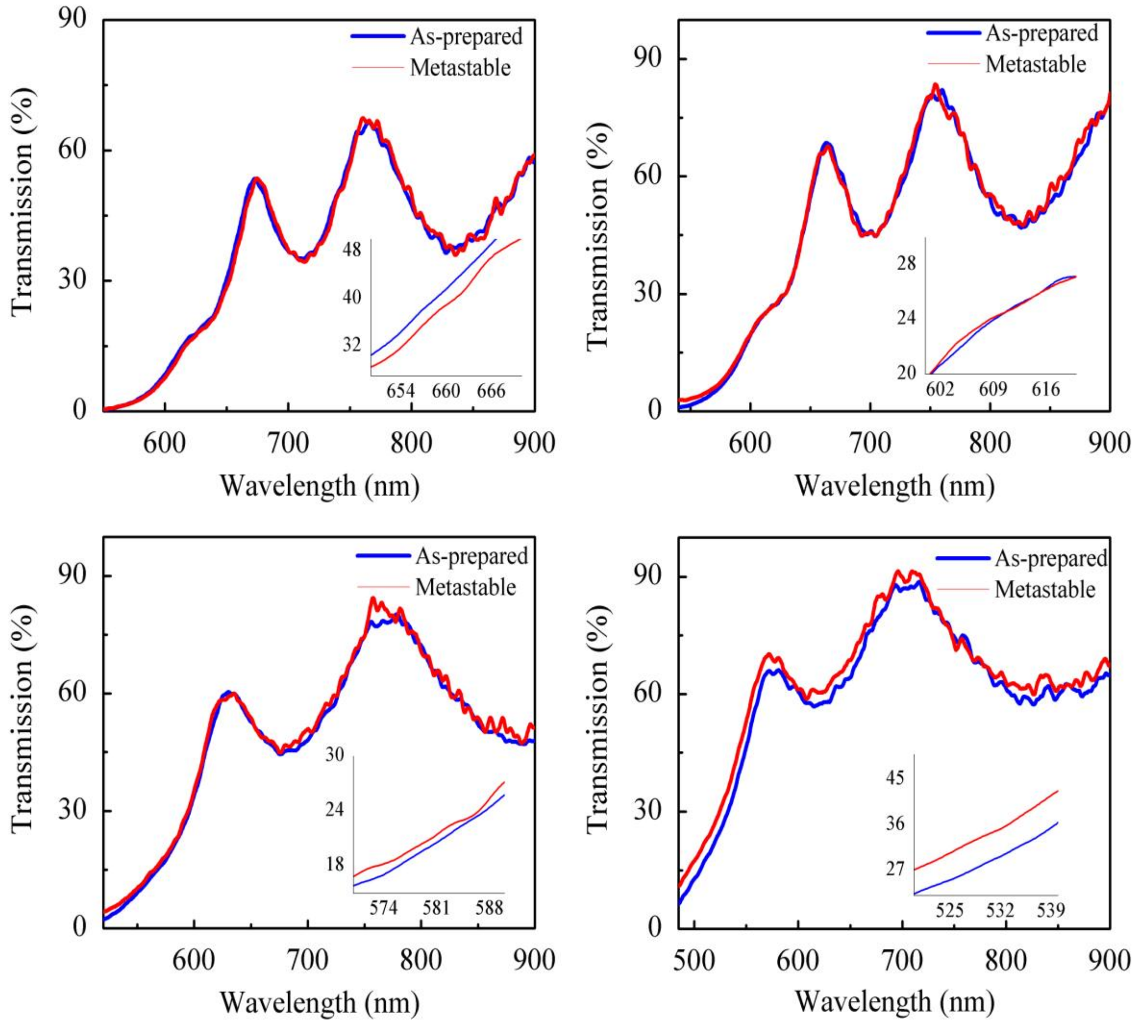

Figure S4. Transmission spectra of (a) a-$Ge_5As_{30}Se_{65}$ (b) a-$Ge_{10}As_{25}Se_{65}$ (c) a-$Ge_{15}As_{20}Se_{65}$ (d) a-$Ge_{25}As_{10}Se_{65}$ thin films in as-prepared and metastable state (after turning off the pump beam). Inset shows expanded region near to bandgap.
